# Multi-omics characteristics of tumor-associated macrophages in the tumor microenvironment of gastric cancer and their exploration of immunotherapy potential

**DOI:** 10.1038/s41598-023-38822-2

**Published:** 2023-10-25

**Authors:** Feng Cao, Yanwei Liu, Yunsheng Cheng, Yong Wang, Yan He, Yanyan Xu

**Affiliations:** 1 Department of General Surgery, University Hospital RWTH Aachen, 52074 Aachen, Germany; 2 Department of General Surgery, The Second Affiliated Hospital of Anhui Medical University, Hefei, 230022 China

**Keywords:** Cancer, Computational biology and bioinformatics, Genetics, Immunology

## Abstract

The incidence and mortality rate of gastric cancer (GC) have remained high worldwide. Although some progress has been made in immunotargeted therapy, the treatment effect remains limited. With more attention has been paid to the immune potential of tumor-associated macrophages (TAMs), but the specific mechanisms of tumor immunity are still unclear. Thus, we screened marker genes in TAMs differentiation (MDMs) through single-cell RNA sequencing, and combined with GC transcriptome data from TCGA and GEO databases, the clinical and TME characteristics, prognostic differences, immune infiltration, and drug sensitivity among different subtypes of patients with GC in different data sets were analyzed. A prognostic model of GC was constructed to evaluate the prognosis and immunotherapy response of patients with GC. In this study, we extensively studied the mutations in MDMs such as CGN, S100A6, and C1QA, and found differences in the infiltration of immune cells and immune checkpoints including M2 TAMs, T cells, CD274, and CTLA4 in different GC subtypes. In the model, we constructed a predictive scoring system with high accuracy and screened out key MDMs-related genes associated with prognosis and M2 TAMs, among which VKORC1 may be involved in GC progression and iron death in tumor cells. Therefore, this study explores the therapeutic strategy of TAMs reprogramming in-depth, providing new ideas for the clinical diagnosis, treatment, and prognosis assessment of GC.

## Introduction

Gastric cancer (GC) is a highly invasive, heterogeneous malignancy that is characterized by insidious onset, obscure clinical symptoms, and rapid progression^1^. Statistically, about 1 million new cases and 800,000 deaths are reported every year^2^. In recent years, although anti-cytotoxic T-lymphocyte associated protein 4 (anti-CTLA-4), anti-programmed cell death-1/programmed cell death-ligand 1 (anti-PD-1/PD-L1) antibody, chimeric antigen receptor (CAR) T-cell therapy and other targeted drugs have achieved certain clinical effects, but most patients with advanced GC still have low prognostic benefits, and the median survival is still less than 12 months^3,4^. Several studies have suggested that this is mainly related to tumor heterogeneity caused by tumor microenvironment (TME)^5,6^.

TME is mainly composed of tumor cells, immune cells, fibroblasts, endothelial cells, and other cells which form an ecosystem allowing the survival of tumor cells. These cellular interactions in TME is a major cause of tumor heterogeneity and contribute to tumor progression and drug resistance^7,8^. Immune cells mainly regulate the TME for tumor growth and progression, which not only provides anti-tumor immunity but also promotes tumor immunity^9^. Numerous studies have demonstrated that tumor cells can be re-programmed by editing the immune cells in the TME to evade immune monitoring and promote tumor cell proliferation and migration^10–12^. However, TAMs are the most dominant immune cell type in the TME^13^. They are involved in TME modification, tumor cell growth, invasion, and metastasis^14^. Previous studies suggest that TAMs are highly flexible and perform various functions at different stages of the TME^15^. In the initial stages of a tumor, they exert their anti-tumor function by killing tumor cells through direct action and the indirect activation of other immune cells. If the tumor continues to progress to advanced stages, the TME will change within the tumor and TAMs will exhibit immunosuppressive function by promoting tumor progression^16^. Chen et al. have found that chitinase 3-like protein 1 (CHI3L1) secreted by M2-TAMs can promote GC transfer through mitogen-activated protein kinase signaling pathway^17^. Although many studies have shown that TAMs play an important role in GC progression, no specific molecular targets and no effective drugs that target TAMs have been found. Compared to targeted lymphocyte therapy strategies, TAMs have the advantage of indirectly activating other immune cells through antigen presentation and directly killing GC cells. In addition, they also accumulate in larger numbers in the TME and can penetrate the dense interstitial tissue around the GC cells. Moreover, compared to GC cells, TAMs are more genetically stable and less susceptible to drug resistance. Therefore, targeting TAMs may be important for identifying GC cell neoantigens and the development of effective immunotherapeutic targets, and in the detection and prognosis assessment of GC.

In this study, we first screened out the key prognostic genes related to the differentiation of TAMs according to GC single-cell RNA sequencing (scRNA-sequencing), and GC tissue transcriptome data to construct a risk model for predicting prognosis and response to immunotherapy. Based on this, we also performed cluster analysis on patients with GC, identified different subtypes of TAMs, and analyzed clinical characteristics, immune infiltration, and differences in TMEs in different subtypes that provide potential markers and targets for GC detection and treatment.

## Methods and materials

### Data source

We downloaded 850 GC samples from The Cancer Genome Atlas (TCGA) (https://cibersortx.stanford.edu/) and the Gene Expression Omnibus (GEO) (https://www.ncbi.nlm.nih.gov/geo/) databases, thus including 433 GC samples from the GSE84437 dataset and 10 single-cell samples from the GSE112302 dataset. All raw files were normalized and annotated by R language. Then, batch effects of the three datasets were eliminated by the "Combat" algorithm^18^, and any missing total survival time (OS) data were excluded.

### Patient population and tissue specimens

Eighty patients diagnosed with GC in the Second Affiliated Hospital of An Medical University were included in the clinical study. Clinical data, such as sex, age, tumor size, stage, lymph node metastasis and activated partial thromboplastin time (APTT), and postoperative tissue samples were collected. All patients and their families have agreed and signed informed consent for this study. And the study was approved by the Ethics Committee of the Second Affiliated Hospital of Anhui Medical University. And the research was performed in accordance with relevant guidelines/regulations and the Declaration of Helsinki.

### Data quality control

We installed the “Seurat” package^19^ in R to filter any sequencing data in GSE112302. First, load the data into the data structure of "Scanpy" using the "read" function of "Scanpy". Then, For each cell, the quality control (QC) metrics were calculated using “perCellQCMetrics” from the “ScatterDensity” package^20^. Performing quality control (QC) and selecting cells using the “CreateSeuratObject” algorithm^19^. Filter conditions included at least three cells, at least 50 genes expressed in each cell, and > 5% mitochondria. The "LogNormalize" method is globally used to scale and normalize the gene expression of each cell^19^.

### Cell annotation and pseudotime analysis

Firstly, Logarithmic conversion and batch effect removal were performed for expression levels. Based on scaled data, the JackStraw algorithm was used for principal component analysis (PCA) and dimension reduction (dims = 1:15)^21^. We used the FindClusters and the FindNeighbor function to perform t-distributed stochastic neighbor embedding (tSNE) clustering on the data of first 15 principal components (PC) (resolution = 0.5). Among them, The “pp.neighbors” function calculates similarities between cells, and the “tl.leiden” function divides cells into clusters. Then, Use the "tl.umap" and "tl.tsne" functions to embed cells in a low-dimensional space. We next annotated and visualized each cluster. The FindMarkers function and the “CIBERSORT” algorithm (https://cibersortx.stanford.edu/) were used to screen for the marker differentials. Further, cell trajectory analysis of both cell and gene annotations was performed using the "monocle" package^22^.

### Analysis of mutations and copy number variations

Macrophage differentiation markers (MDMs) were extracted form sc-RNA sequencing analysis, and their mutations in GC samples from the TCGA database were calculated using the R language’s "maftools" package^23^, and a waterfall chart of MDMs mutations was created. Copy number data in GC downloaded from the UCSC Xena (https://xena.ucsc.edu/) database to analyze any copy number variations (CNV) of MDMs by "Rcircos" of the R language (https://github.com/hzhanghenry/RCircos).

### Enrichment analysis

A fold-change of ≥ 1 and an adjusted *P*-value of < 0.05 were screened according to MDMs. Gene ontology (GO) and Kyoto encyclopedia of genes and genomes enrichment analysis (KEGG) functional enrichment analyses of MDMs and MDMs-related genes were then performed using the "enrichplot" and "org.Hs.eg.db" packages to explore the underlying biological role of genes, comprising three terms, i.e.,biological process (BP), cellular component (CC), molecular function (MF) and biological pathways^24–26^. In addition, after downloading the signaling pathway set file from the MSigDB database immunological enrichment analyses of MDMs and MDM-related genes were performed using the “GSVA” packages^27^. In addition, a single-sample gene set enrichment analysis (ssGSEA) algorithm^28^ was used to quantitatively analyse each cell to compare differences in immune cell infiltration among different subtypes.

### Consensus clustering analysis

Both “Reshape2” and “ggpubr” were first used to extract MDMs that were differentially expressed in both normal and tumor tissues of patients with GC. Combined with clinical prognostic information for each sample, those MDMs with significant survival differences were further screened and their interaction network was mapped by “igraph” and “psych” algorithm. “RColorBrewer” package was used to calculate the importance of all nodes and identify the key nodes^29^. Based on the extent of their expression in each sample, a consensus unsupervised cluster analysis was conducted for all patients with GC using the k-means method in the "ConensusClusterPlus" package to classify all GC patients into different molecular subtypes^30^. The principal component analysis (PCA) graph was drawn by analyzing the expression levels of MDMs and visualized by the "ggplot2" software package^31^.

### Analysis of clinical characteristics and survival differences

We analyzed the clinical characteristics and prognosis of patients with GC in different subtypes based on the results of the consensus unsupervised cluster. The "ggplot2 3.4.0" package^31^ was used to compare age, gender, and the TNM staging of all patients across all subtypes, The prognostic and clinical features between distinct subtypes were compared using the R "survival" package and the "PheATmap" package^32^, and was visualized with the R language. In addition, we incorporates expression data and clinical information of MDMs genes by “limma” package. Kaplan–Meier survival curves for survival time and state of patients with GC with different subtypes were analyzed and plotted by “survival” and “survminer” packages.

### Screening for prognostic-related DEGs

Univariate COX regression analysis was performed for MDMs related differentially expressed genes (DEGs) in both "limma" and "survival" packages to obtain prognostic genes in the TCGA database. Prognostic-related genes in the GSE84437 dataset were detected by the "WGCNA" package^33^. During this process, the shear height was set at 1000; the cut size, at 10; the number of module genes was ≥ 30; and the clustering height of module characteristic genes was 0.3. The prognostic genes obtained in both data sets were selected through the "VennDiagram" package to take intersection genes and a Venn diagram was drawn.

### Constructing a prognostic model and prognostic scoring system

The "caret" package (https://github.com/topepo/caret/) was used to divide patients with GC (1:1) into training and test groups to construct and verify models, respectively. The packages "glmnet", "survminer" and "timeROC" were then used for the least absolute shrinkage and selection operator (LASSO), and the multivariate COX regression analysis was used for prognostic associated DEGs^34,35^. The risk scores (RS) of genes with non-zero regression coefficients were calculated. RS = $\sum_{j=1}^{n}Xj*Coefj$, where n represents the number of included genes, $Xj$, the gene expression, and $Coefj$ represents the risk coefficient. The independent prognostic analysis function was defined using the package "survival" to confirm that the RS score was an independent factor that affected patient prognosis, and a forest map was drawn subsequently. The "SurvivalROC" package was used to plot and calculate the area under the curve (AUC) of the time-dependent receiver operating characteristic (ROC) to evaluate the predictive power of this prognostic model. We then used the "rms" package and the "calibration" function to draw a nomogram diagram and a calibration curve.

### Immune-infiltration and TME analysis

The "preprocessCore" package was used to calculate the degree of infiltration of various immune cells and immune checkpoints in different samples, and the "CIBERSORT" analysis method was used to calculate the difference in immune cells and immune checkpoints and the correlation of DEGs different subtypes between high and low risk groups. For TME analysis, differences in stromal, immune, and estimation scores were calculated. Any difference in tumor mutation burden (TMB) was then analyzed using "reshape2" and "ggpubr" packages after combining them with TMB data and RS scores of patients with GC. Finally, combined with the microsatellite instability (MSI) data and tumor stem cells (CSCs) scoring data of all tumors, the correlation between MSI and CSCs and RS was calculated through the "ggplot2" and "ggpubr" packages^31^.

### Drug susceptibility analysis

The tumor chemotherapy drug data was downloaded from the genomics of drug sensitivity in cancer (GDSC) (http://www.cancerrxgene.org/) database. A formula package of "pRophetic" was used to calculate the IC50 value for GC chemotherapy drugs. The difference in IC50 between the high-risk and low-risk groups was compared using the Wilcoxon test, and the filtering condition was set as *P* < 0.001.

### Quantitative real-time PCR

Total RNA was extracted from tumor tissues and normal tissues of 8 randomly selected pairs of GC patients after operation using a Fast Pure Cell/Tissue Total RNA Isolation Kit (ES science, China). Then, we reverse transcribed the RNA to cDNA under standard conditions with Evo M-MLV RT Premix (Accurate Biotechnology, China). The SYBR Green PCR kit (accurate biotechnology, China) was used for quantitative real-time PCR. We selected GAPDH as the internal control. Relative expression levels of target genes were calculated as 2^-ΔCt^. The primer of target gene as follows:

VKORC1: F, GAGCCTGATGTGGCTCAGTT

               R, TCAGTGCCTCTTAGCCTTGC

### Statistical analyses

The study was carried out under the R version 4.1.3 (https://www.r-project.org/), Strawberry Perl version 5.32.1.1 (https://strawberryperl.com/) and GraphPad Prism 7 (https://www.graphpad.com/). The data are expressed as the means ± SD, and Student’s t-test or one-way ANOVA test was used for difference determinations. *, **, *** indicated *P* < 0.05, *P* < 0.01, *P* < 0.001, respectively. A *P* < 0.05 was considered statistically significant.

### Ethics approval and consent to participate

The study was approved by the Ethics Committee of the Second Affiliated Hospital of Anhui Medical University.

## Results

### Screening of MDMs

**Figure 1:**
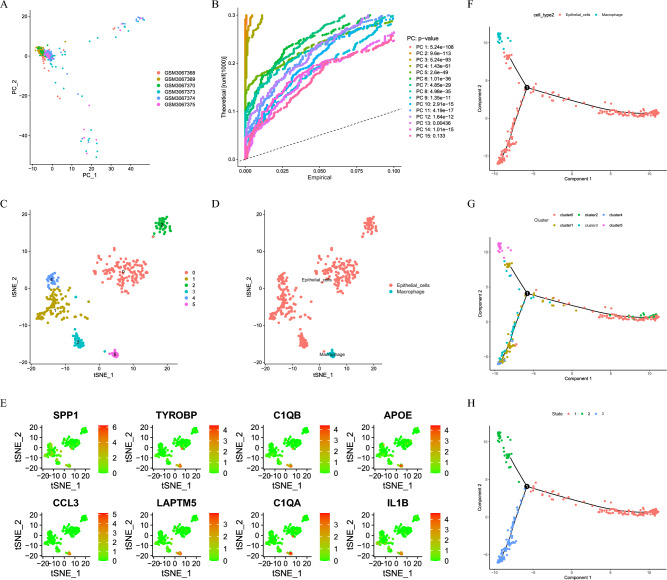
Analysis of scRNA sequencing. (**A, B**) PCA clustering of the genes expression matrix. (**C, D**) Cell clusters and annotates for GSE112302 of GC patients. (**E**) Expression pattern of top 8 marker genes in macrophage cluster. (**F–H**) cell trajectories analysis.

Our study flowchart has been described in Figure S1. Before proceeding with analyzing our RNA-sequencing data, a thorough quality control (QC) of all data is required to filter out the influence of low-quality cells and mitochondrial genes (Figure S2). The total number of unique molecular identifiers (UMI) in each cell does not correlate with mitochondria, and the correlation with the total number of genes is 0.38 (Figure S2B). Dimension reduction analysis is performed on scRNA-sequencing data, and PCA showed that GC cells showed a significant separation trend (Fig. 1A). After normalization, 14 PCs were screened (*P* < 0.05) (Fig. 1B). The GC cells were then divided into six clusters by further performing a tSNE cluster analysis (Fig. 1C). By using cell marker annotation, it was found that the clusters were mainly composed of epithelial cells and macrophages (Fig. 1D). Among them, SPP1, TYROBP, C1QB, APOE, CCL3, LAPTM5, C1QA, and IL1B were the top 8 genes that had the highest expression in the macrophage cluster (Fig. 1E). In addition, pseudotime analysis showed that cells in the six clusters differentiated into 3-medium states and were eventually differentiated into macrophages in the second branch (Fig. 1F–H). Finally, 54 MDMs were obtained through the intersection of genes in the second branch, marker genes in macrophages, and differentially expressed genes in tumor tissues.

### Genetic and transcriptional characteristics of MDMs

**Figure 2:**
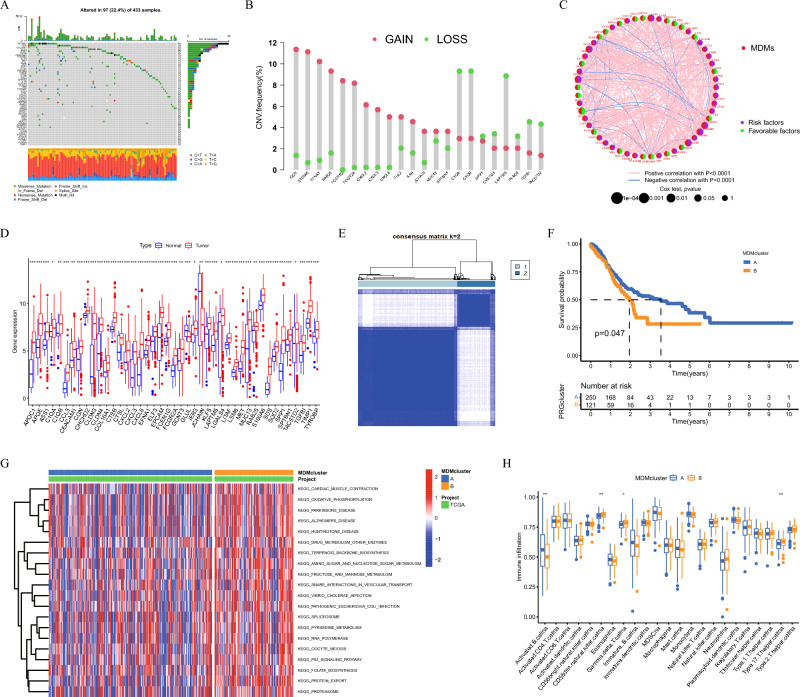
Genetic and transcriptional analysis in TCGA dataset. (**A**) Mutation frequencies and types of MDMs. (**B**) CNV of MDMs. (**C**) The interaction network between prognostic related MDMs. (**D**) Differential expression of MDMs. (**E**) Unsupervised clustering of MDMs and consensus matrix heatmaps for k = 2 base on TCGA datasets. (**F**) Kaplan–Meier survival curves. (**G**) GSVA enrichment analysis. (**H**) Immune cell infiltration characteristics.

The results from the mutation analysis showed that 22.4% of 433 GC samples have mutations in MDMs that are dominated by nonsense mutations. The five MDMs with the highest mutation frequency are SPTBN1, CGN, COL16A1, ELF3, and ASS1 (Fig. 2A). In addition, during analyzing CNVs of MDMs, it was found that 22 MDMs had significant CNVs, among which CGN, S100A6, and EFNA1 showed obvious amplification, and C1QA, C1QB, and LAPTM5 showed extensive deletion (Fig. 2B).

### Prognostic characteristics and molecular subtypes of MDMs

The expression characteristics of 54 MDMs in tumors and normal tissues were verified in the TCGA data. We found that 42 MDMs were highly expressed in tumors, and 37 MDMs were prognostic factors for GC (Figs. 2C,D). Interestingly, CGN, S100A6, and EFNA1 with significant CNV amplification were favorable prognostic factors, and C1QA, C1QB, and LAPTM5 with widespread deletion were risk factors. This suggests that the role of MDMs in GC progression and prognosis could be related to their respective CNV. In addition, GO enrichment analysis showed that these MDMs were mainly enriched in biological processes such as cellular lipopolysaccharide reaction and cellular immune response, and had molecular functions of activating cytokines and chemokines (Figure S3A). The KEGG analysis was mainly concentrated on cytokine-mediated signaling pathways and regulatory pathways of epithelial cell migration (Figure S3B).

### Clinical and immunoinvasive characteristics of different molecular types of MDMs

**Figure 3:**
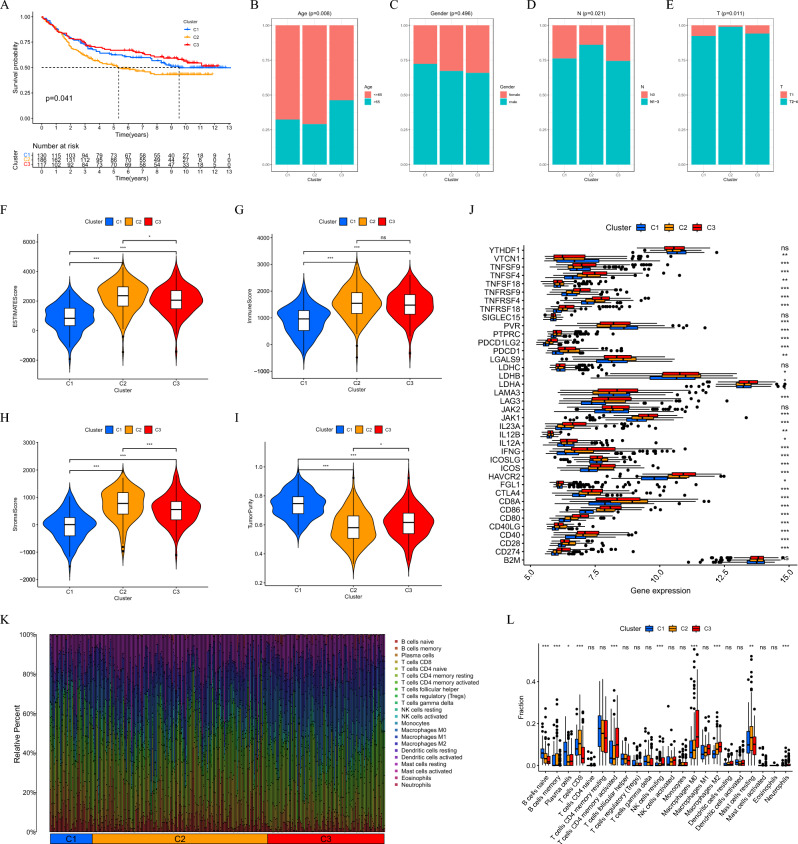
Clinical characteristics and TME analysis of MDMs in GEO datasets. (**A**) Survival curves in three subtypes. (**B–E**) Differences of clinical characteristics. (**F–I**) Differences of the TME Score and tumor purity between the three subtypes. (**J**) Differential expression of immune checkpoints. (**K–L**) immune cell infiltration analysis.

Unsupervised cluster analysis showed that all patients in the TCGA data set could be divided into subtypes, A and B, and the Kaplan–Meier curve showed survival differences (*P* < 0.05) (Figs. 2E,F). In GSVA enrichment analysis, subtype B with poor prognosis was mainly enriched in oxidative phosphorylation, substance metabolism, and the P53 signaling pathways (Fig. 2G). The B cells, natural killer cells and type 17 T helper cells were all highly infiltrated in the B subtype (Fig. 2H). In addition, in the GSE84437 data set, patients with GC could be divided into three subtypes (Figure S3C). According to MDMs expression, and a significant difference in their prognosis was observed (*P* < 0.05) (Fig. 3A). In terms of clinical features, there was a significant difference in age and T- and N-stages among the three subtypes except for gender (*P* < 0.05) (Fig. 3B–E). The C2 subtype patients had the worst prognosis and the highest degree of TME mesenchymal cell and immune cell infiltration with the lowest tumor purity (Fig. 3F–I). Among immune cells, the proportion of macrophages and T cells was the highest. However, the degree of infiltration for M0 and M2 macrophages in the C3 subtype was significantly higher than that in the other two subtypes (Fig. 3K–L). Moreover, several immune checkpoints, such as CD274, CD80, and CTLA4, were significantly up-regulated in the C3 subtype (Fig. 3J). Survival analysis found differences in the survival time between these high- and low-infiltrating immune checkpoints and immune cells (Figures S4–S5). This suggests that immune cells in the TME may shift to immunosuppressive phenotypes, and play a pro-tumor function during tumor progression.

### The classification of prognostic-related DEGs

**Figure 4:**
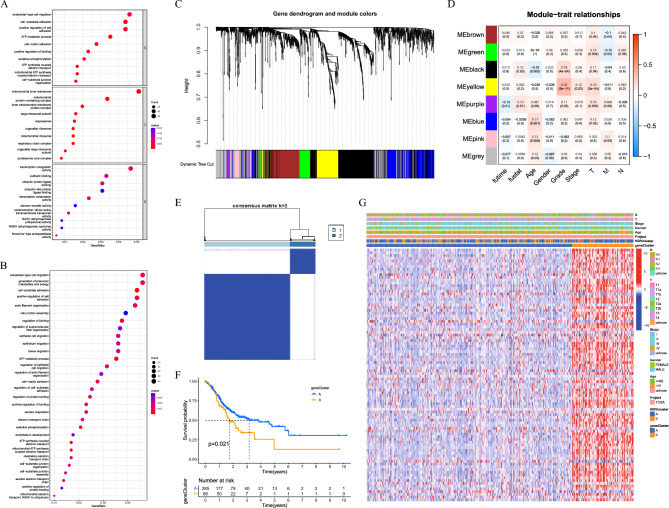
Screening MDMs related genes associated with prognosis and calculating risk score. (**A, B**) GO and KEGG enrichment analyses of MDMs related genes. (**C**) WGCNA network module clustering tree. (**D**) Clustering module characteristics, Correlation between modular genes and clinical features. (**E**) Unsupervised clustering analysis of MDMs related genes and consensus matrix heatmaps for k = 2 base on TCGA and GEO datasets. (**F**) Kaplan–Meier survival curves. (**G**) Difference of MDMs related genes expression in distinct clinical characteristic groups.

We screend1661 MDM-related DEGs, and enrichment analysis of these DEGs showed that they were mainly involved in energy metabolism and cell adhesion processes (Fig. 4A,B). Further, weighted correlation network analysis (WGCNA) showed that two gene modules were associated with prognosis with 540 DEGs (Fig. 4C,D). Univariate COX analysis showed that 129 DEGs were prognostically correlated, and 92 prognostic correlated DEGs were obtained at the intersection of the WGCNA and Univariate COX analysis (Figure S6A). And the clinical characteristics and prognosis of patients with subtype A were better than those of subtype B (Fig. 4E,G).

### Establishment of the prognostic model

**Figure 5:**
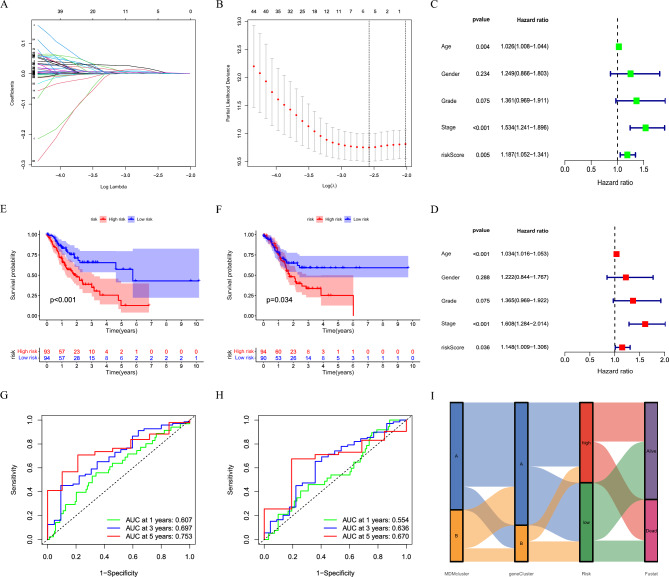
Construction of prediction model. (**A, B**) LASSO regression analysis and partial likelihood deviance. (**C, D**) Univariate and multivariate COX regression analysis of risk score. (**E, F**) Kaplan–Meier survival curves in distinct risk groups. (**G, H**) ROC curves. (**I**) Alluvial diagram of subtype distributions.

We analyzed 92 prognostic-related DEGs by the LASSO regression, and the risk coefficient was calculated, and five prognostic-related DEGs were further screened out (Fig. 5A,B). Then, three DEGs with the best predictive value were selected. In COX regression analyses of RS, it was found that RS was an independent prognostic factor (Fig. 5C,D). After patients were divided according to the median value of RS, significant differences in survival times between the two groups were noted (Fig. 5E,F). In addition, the AUC of the ROC, which measures the ability of RS to predict the prognosis was higher than 0.6 (Fig. 5G,H). Thus, the survival status of patients could be comprehensively assessed according to the MDM classification, DEGs classification, and RS (Fig. 5I).

### Characteristics of prognostic models and nomogram prediction system

**Figure 6:**
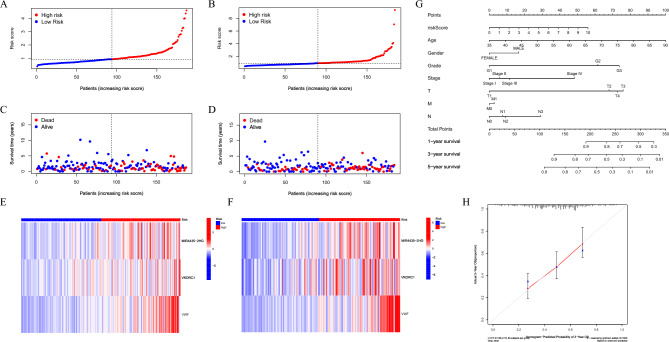
Prognostic value of the MDMs related genes signature and construction of nomogram. (**A, B**) RS distribution. (**C, D**) Survival status. (**E, F**) Heatmap of the 3 hub genes. (**G**) Nomogram scoring system. (**H**) Calibration curves.

Sorting patients with GC according to the RS value showed higher mortality (Fig. 6A–D). Moreover, DEGs, the key to RS score calculation, were also highly expressed in the high-risk group (Fig. 6E–F). This indicated that our prognostic model has a good ability to evaluate clinical features. Therefore, this study constructed a nomogram prediction system(Fig. 6G). The calibration diagram and ROC curve was also used to verify the accuracy of our prediction system (Fig. 6H and Figure S8).

### Correlation between RS, immune cells, and somatic mutations

**Figure 7:**
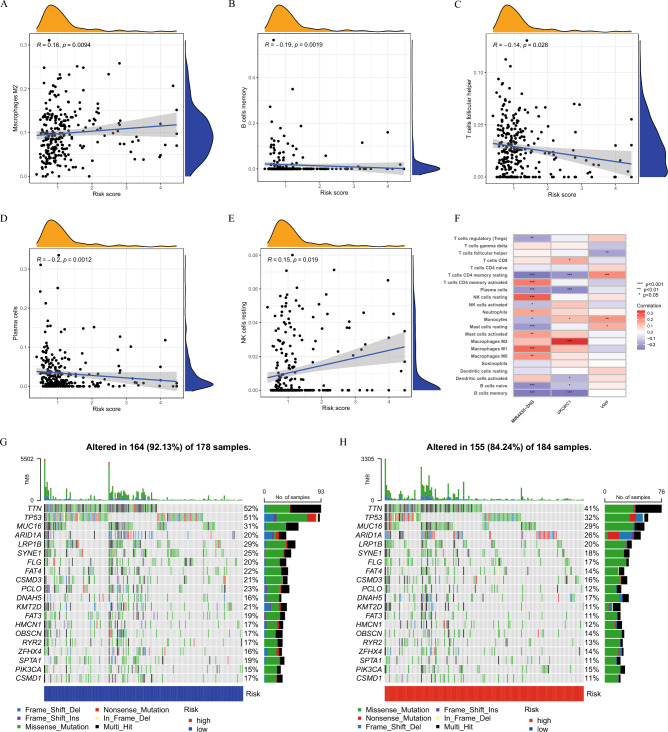
Immune cell infiltration and mutation analysis. (**A–E**) Correlation between immune cells and RS. (**F**) Correlations between immune cells and 3 hub genes. (**G, H**) Mutation of genes in distinct RS groups.

Correlation analysis showed that RS was positively correlated with M2 macrophages and NK cells, and negatively correlated with memory B cells, follicular helper T cells, and plasma cells (Fig. 7A–E). Surprisingly, key DEGs also showed a strong correlation with macrophages, among which VKORC1 gene had the highest correlation with M2 macrophages (Fig. 7F). In addition, in the somatic mutation analysis, the median somatic mutation was found to be slightly higher in the low-risk group. Genes with the highest mutation frequency were TTN, TP53, MUC16, ARID1A, and LRP1B, which suggested that somatic mutation and gene instability are negatively associated with RS (Fig. 7G–H).

### The difference in TME, TMB, MSI, and drug sensitivity

**Figure 8:**
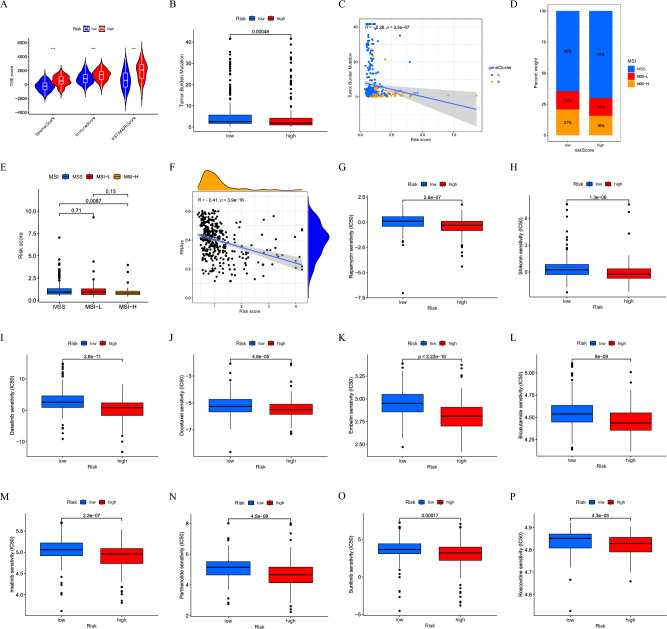
Analysis of TME characteristics and drug susceptibility. (**A**) Differences of the TME Score. (**B, C**) Correction between TBM and RS. (**D, E**) Correction between MSI and RS. (**F**) Correlation of RS with CSCs. (**G–P**) The differences of chemotherapeutic sensitivity.

The TME analysis showed that stromalScore, immuneScore, and the ESTIMATE Score in the high-risk group were significantly higher than in the low-risk group (Fig. 8A). On the contrary, the TMB was negative correlation with RS (Fig. 8B,C). In addition, RS was correlated with the MSI status (Fig. 8D,E). Furthermore, an obvious negative correlation between RS and CSCs in the correlation analysis of CSCs was also reported (Fig. 8F). These results indicated that GC cells with lower RS were more sensitive to immunotherapy and have less differentiated stem cell characteristics. Finally, we evaluated the correlation between RS and GC chemotherapy drug sensitivity and found a significant difference between the semi-inhibitory concentration (IC50) of multiple chemotherapy drugs in both groups. Among them, IC50 of rapamycin, shikonin, dasatinib, docetaxel, embelin, bicalutamide, imatinib, parthenolide, sunitinib, and roscovitine was lower in the high-risk group (Fig. 8G–P).

### VKORC1 was up-regulated in GC and associated with tumor progression and survival

To further verify the role of VKORC1 in GC. We examined its mRNA level in 8 pairs of GC tissues and found that the expression level of VKORC1 was significantly upregulated than that in matched normal tissues (*P* = 0.01) (Figure S7A). Then, we next analysed the correction of VKORC1 expression level and the parameters of 80 GC patients. In the prognostic analysis, both the prognostic information from the TCGA public database or from our clinical research, the results showed that patients with high VKORC1 expression had poor OS (Figure S7B–C). In additional, Further analysis showed that VKORC1 was significantly associated with tumor size (*P* = 0.003), stage (*P* = 0,006), lymph node metastasis (*P* = 0.001), and APTT (*P* = 0.015), but not with sex (*P* = 0.528) and age (*P* = 0.681) (Figure S7D–I). Interestingly, VKORC1 expression decreased significantly in advanced tumors. This may be one of the reasons why patients with advanced tumors have poor clotting function.

## Discussion

In recent years, lymphocytes have been the focus of targeted therapy for malignant tumors, and the immunotherapeutic potential of TAMs have not been studied extensively. Although TAMs are highly flexible and can have both functions of promoting immunity and suppressing immunity in the TME, they mainly exhibit immunosuppressive phenotypes in advanced GC. Therefore, targeting TAMs-specific molecular markers to reprogram them into anti-tumor phenotypes is a novel cancer treatment strategy. In the present study, we comprehensively analyzed the multiomics of MDMs obtained through screening in the RNA-sequencing data at the transcriptional and genetic levels, to reveal any mutation and CNVs. different molecular subtypes were identified, and the clinicopathological characteristics, prognosis, and TME differences among the different subtypes were discussed in depth. Identification of risk factors in MDMs and their associated genes contribute to a better understanding of the molecular mechanism of GC, providing new immune strategies and drug targets for clinical treatment. Thus, we also constructed a prognostic scoring system for patients with GC that can accurately evaluate the prognostic survival time of patients.

TAMs are an important component of innate and adaptive immunity and can be polarized into two phenotypes, M1 and M2 according to different signals in TME^36,37^. The M1-type macrophages can kill tumor cells by directly mediating cytotoxic effects and secreting reactive oxygen species and NO molecules^38,39^. In contrast, epithelial growth factors (EGF) and matrix metalloproteinases (MMPs) secreted by the M2-type macrophages can promote the proliferation and metastasis of tumor cells^40,41^. Therefore interaction of TAMs with TME, and targeting TAMs is considered the latest therapeutic strategy for tumors^42^. Some studies suggest that misexpression and ineffectual expression of proteins caused by gene mutation and CNV may be one of the reasons for tumor development^43,44^. Thus, further exploration of mutant genes can lead to a better understanding of the molecular mechanisms of GG. In addition, studies have suggested that mutated genes with high specificity may be useful for the screening and diagnosis of tumors^45^. Ghaffari et al. found that the Baculoviral inhibitor of apoptosis repeat containing 5 (BIRC5) gene with high CNV could be used as a marker for early cancer detection^46^. Gene mutation is a significant proportion of genetic factors associated with cancer. Tumorigenesis and progression are the result of multiple driver gene mutations. Zhang et al.^47^ showed that patients with ATR, BLM and MLH1 mutations in prostate cancer had worse prognosis and increased Olaparib sensitivity. In this study, the degree of Cingulin (CGN) mutation was the most remarkable, and CGN, as a connexin of endothelial cells, was previously thought to be mainly involved in the regulation of the endothelial barrier^48^. However, other studies have shown that CGN can promote cell proliferation by regulating the activity of member A (RhoA) in the Ras homolog gene family^49^. In malignant tumors, Zhang et al. found that abnormal expression or CGN targeting could induce the proliferation and invasion of ovarian and colorectal cancer cells^50,51^. However, the role of CGN in GC is unclear at present.

The three hub MDMs-related gene risk models constructed in this study not only identified the risk degree of different patients with GC but also dynamically monitored the tumor progression and prognosis more accurately. Similar studies have been conducted previously when they screened potential target of endometriosis and constructed a risk model of RNA-binding proteins (RBPs) to predict patient prognosis with renal papillary cell carcinoma (pRCC)^52,53^. And, Liu et al.^54^ deeply analyzed the tumor stemness-related genes in renal clear cell carcinoma (ccRCC), and identified a key gene that was significantly associated with the prognosis of ccRCC and promoted the proliferation of ccRCC cells. Compared to whole transcriptome sequencing, the quantitative detection of key gene expression levels also has higher accuracy in clinical diagnosis, treatment, and prognosis assessment, and is more economical and clinically feasible. In addition, M2 macrophages mainly infiltrate in tumor tissues of patients with better prognosis and M2 macrophages had a higher positive correlation with the RS of this model. In advanced GC, interleukin(IL)-6 and IL8 released by mesenchymal stromal cells (MSCs) promote polarization of M2-type TAMs in TME via the JAK2/STAT3 signaling pathway, resulting in immunosuppressive TME^55^. M2 TAMs activation is regulated by a variety of signaling pathways. This study indicated that the PI3K/Akt/mTOR pathway mediated by various molecular signals is involved in the regulation of M2 TAMs^56^. Proinflammatory cytokines (TNF-α, IL-β, and IL-23) released by M2-type TAMs in solid tumors continue induce overexpression of PD-L1, CTLA4, and glucocorticoid-induced TNF receptor family-regulated protein (GITR) to inhibit tumor immunity^57^. However, these cytokine induced cascades are closely associated with macrophages. Activated NF-κB, nuclear factor erythroid 2-related factor 2 (Nrf2), TGF-β/Smad, The MAPK and JAK/STAT signaling pathway in turn promote the polarization and infiltration of M2 macrophages, thus promoting the progression of the disease^58^. One of the most important factors in tumor progression is the formation of new blood vessels due to hypoxia. M2-type TAMs mainly accumulate in this area to regulate the secretion of vascular endothelial growth factor (VEGF)^59^. M2 TAMs can activate Nrf2 signaling pathway in cancer cells, in turn, to increase cancer cells epithelial-mesenchymal transition (EMT) through paracrine VEGF^60^. Thence, studies have found that targeted VEGF inhibition can reduce TAMs infiltration and cytokine secretion, thus effectively inhibiting the growth and proliferation of tumors^61^. In addition, TAMs are an integral component of the hematogenous spread and metastasis of tumor cells. Xu et al.^62^ study showed that target MAPK pathway blocking M2 macrophages can effectively inhibit the growth, invasion, migration and angiogenesis of lung cancer. In addition, M2 macrophages can also induce the formation of Tregs by activating the TGF-β/Smad signaling pathway, thus promoting tumor progression^63^. Direct adhesion of TAMs to type IV collagen and eventual increased extracellular matrix remodeling is another reason for promoting tumor cell metastasis^64^. Therefore, all this evidence proves that TAMs promote tumor cell proliferation and invasion in advanced tumors, and play a role in tumor immune suppression.

Reprogramming of TAMs is a new malignant tumor therapy that has been recently proposed. Its principle is to convert M2-type TAMs back into M1-type TAMs to play the role of an antitumor role in TME. Interference with the nuclear factorκB (NF-κB) signaling pathway or association with tumor necrosis factor-α (TNF-α) can result in M1-type TAMs that promote tumor regression^65,66^. The CSF-1/CSF-1R signaling pathway is one of the most important pathways for the reversal of TAMs that can regulate the differentiation of myeloid cells and TAMs^67^. Emactuzumab is a human monoclonal antibody against CSF-1R. In a drug study, Emactuzumab was found to have a higher response rate in patients with tendon sheath giant cell tumors^68^. In addition, tyrosine kinase inhibitors (TKIs) can also inhibit this pathway. In glioma clinical trials, a TKI BLZ945 showed promising results when combined with insulin-like growth factor 1 receptor (IGF1R) and phosphoinositol 3 kinase (PI3K) inhibitors^69^. In addition, the CCL2-CCR2 axis is also an effective way to reverse TAMs^70,71^.In addition to molecular effects, radiation therapy for solid tumors was also found to affect TAMs. One study reported that low-dose gamma irradiation allowed macrophages to differentiate into iNOS + /M1 phenotypes for killing solid tumors^72^. However, until now, no effective targets and drugs have been developed to reverse TAMs in solid tumors. Therefore, we hope to further explore any genetic characteristics of TAMs in GC TME, to provide help for treating patients with GC with TAMs reprogramming strategy.

In this model, we unexpectedly found that the hub gene VKORC1 was highly correlated with M2 macrophages in TME. VKORC1 is the primary functional subunit of vitamin K epoxide reductase (VKOR), which is involved in regulating vitamin K synthesis^73^. Although vitamin K functions primarily as a clotting agent, recent studies have shown that it is also effective in preventing ferroptosis in cells and tissues^74^. In addition, Beaudin et al. also found that VKORC1 and VKORC1L1 were highly expressed in triple-negative breast cancer (TNBC) cell lines and advanced breast cancer tissues, and promoted the growth of TNBC cells, and the expression of glutamate-modified proteins through vitamin K1 synthesis^75^. This suggests that VKORC1 could be involved in regulating the proliferation and in the programmed death of tumor cells through vitamin K. However, specific mechanisms are still unclear, especially about TAMs and immune cells. Therefore, we speculate that VKORC1 may play a role in tumor progression and may also be regulated by TAMs.

## Conclusion

In this study, the multiomic features such as CNV, clinicopathological indicators, prognosis, immune infiltration, and TME of MDMs in GC were analyzed in depth, and a prognostic prediction model integrating multiple molecular markers, clinicopathological parameters, and other multi-level indicators were constructed to further explore a therapeutic strategy of reprogramming TAMs. Our findings show a new direction for clinical diagnosis, treatment, and prognosis evaluation of GC. We also observed that hub gene VKORC1 might be involved in GC progression and iron death of tumor cells.

### Supplementary Information

Supplementary Figure 1.

Supplementary Figure 2.

Supplementary Figure 3.

Supplementary Figure 4.

Supplementary Figure 5.

Supplementary Figure 6.

Supplementary Figure 7.

Supplementary Figure 8.

Supplementary Legends.

## Data Availability

The datasets generated and/or analysed during the current study are available in the TCGA (https://portal.gdc.cancer.gov/), Gene Expression Omnibus (GEO) (https://www.ncbi.nlm.nih.gov/geo/), UCSC Xena database (https://xena.ucsc.edu/) and Genomics of Drug Sensitivity in Cancer (GDSC) database (https://cancerrxgene.org). And all code and original data you can find in the link (https://www.jianguoyun.com/p/DaSrlpIQsPGzCxjt5PYEIAA). Or you can contact authous.
