# Supplementary figures and images for: Multi-omics characteristics of tumor-associated macrophages in the tumor microenvironment of gastric cancer and their exploration of immunotherapy potential
Source: Sci Rep. 2023 Oct 25;13:18265. doi: 10.1038/s41598-023-38822-2 (PMC10600170; doi:10.1038/s41598-023-38822-2)

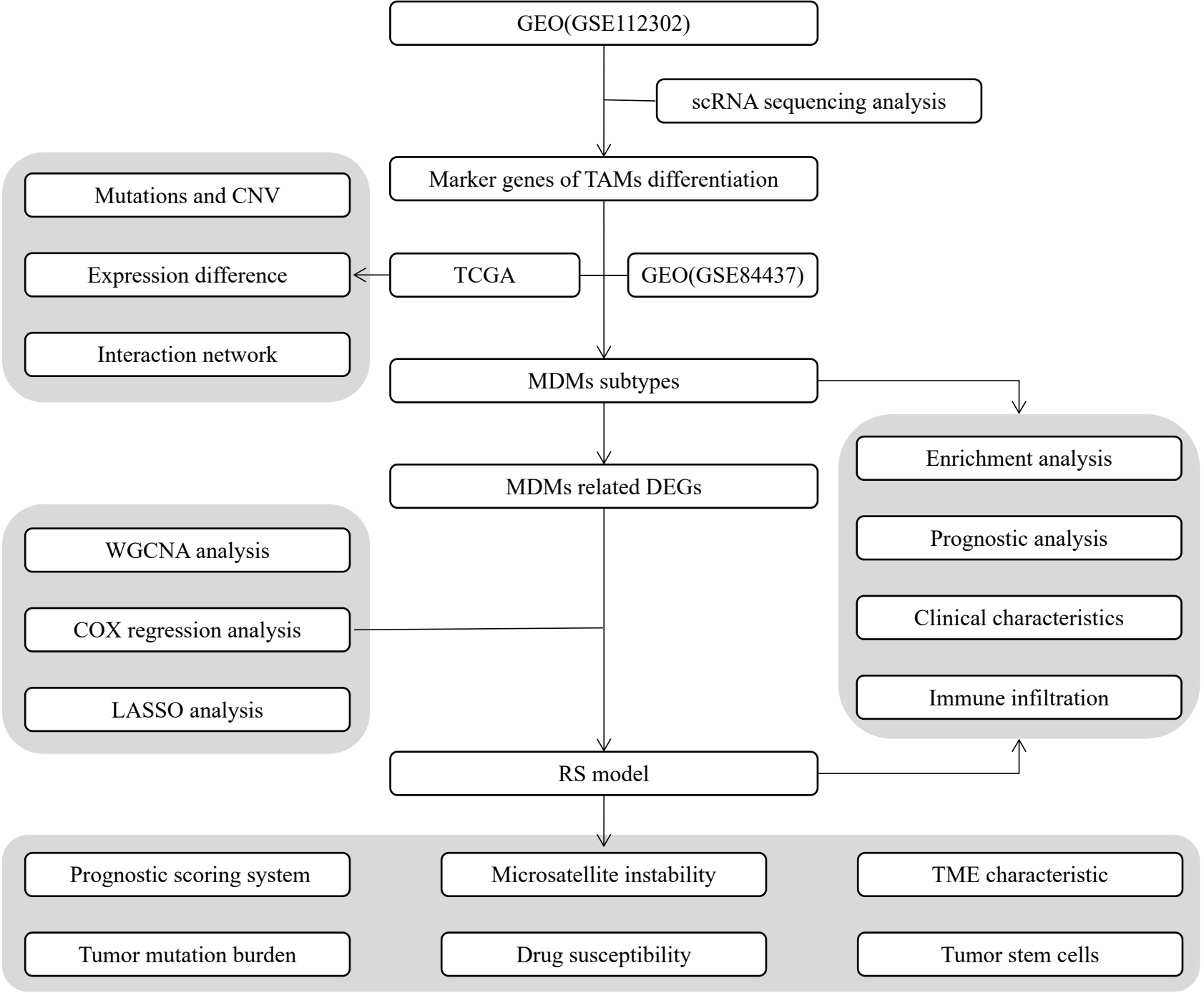

Supplement: Supplementary file 1 — Supplementary Figure 1. [file 41598_2023_38822_MOESM1_ESM.pdf]

A

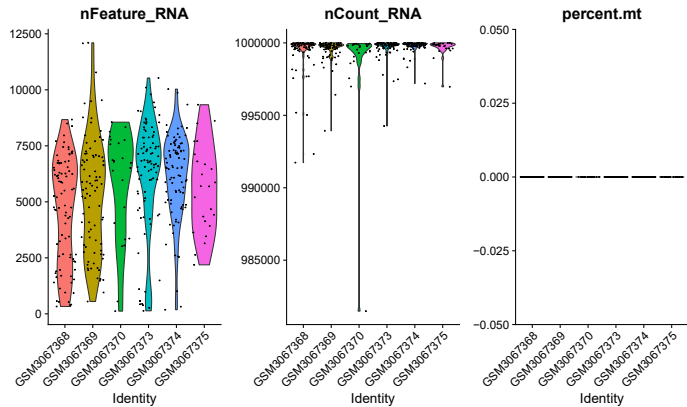

B

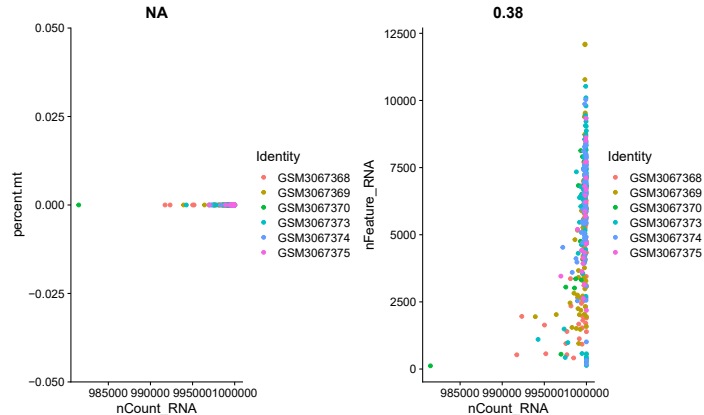

Supplement: Supplementary file 2 — Supplementary Figure 2. [file 41598_2023_38822_MOESM2_ESM.pdf]

A

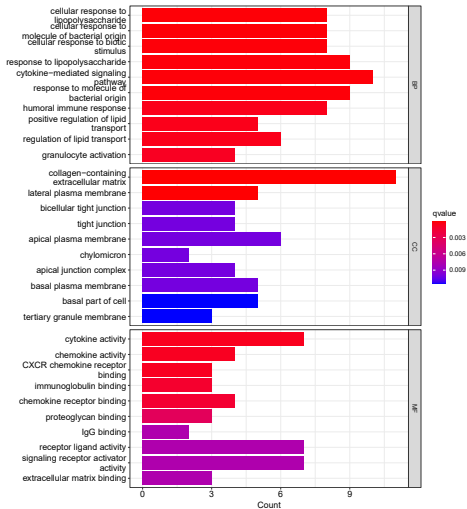

B

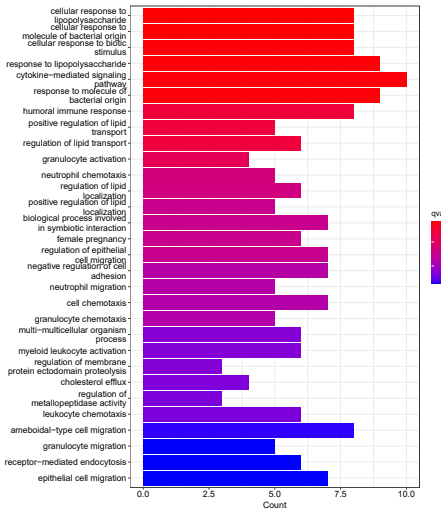

C

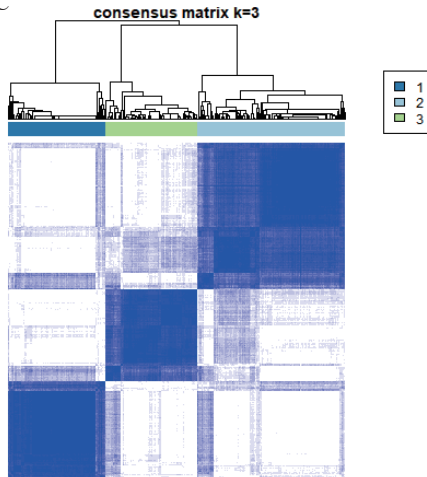

Supplement: Supplementary file 3 — Supplementary Figure 3. [file 41598_2023_38822_MOESM3_ESM.pdf]

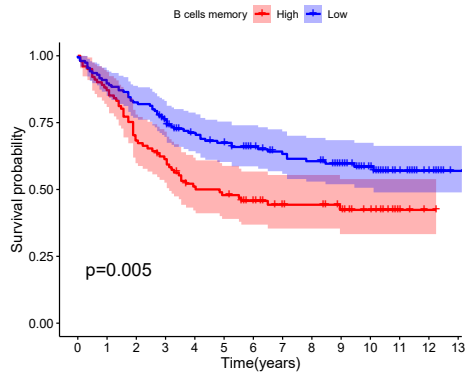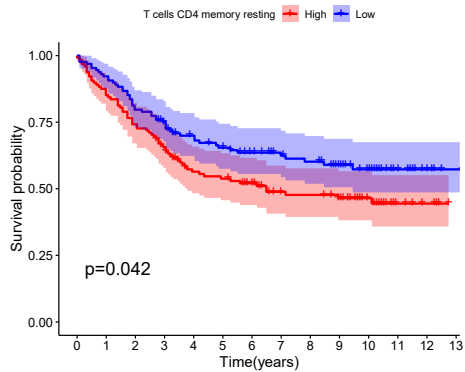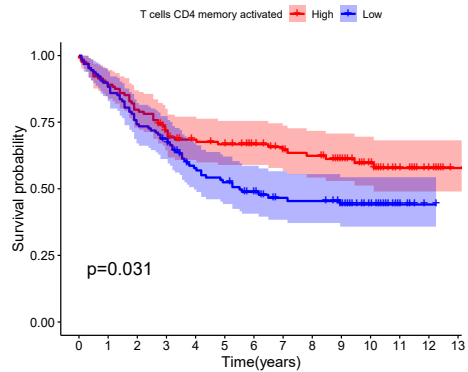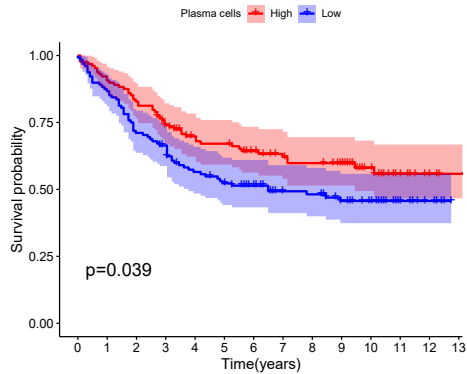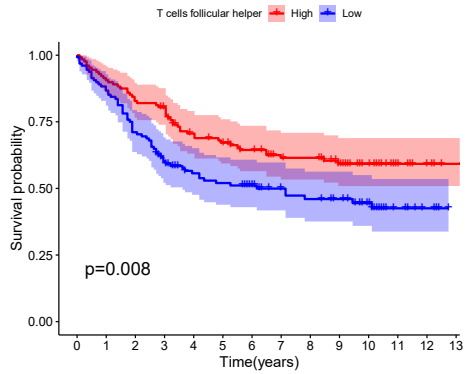

Supplement: Supplementary file 4 — Supplementary Figure 4. [file 41598_2023_38822_MOESM4_ESM.pdf]

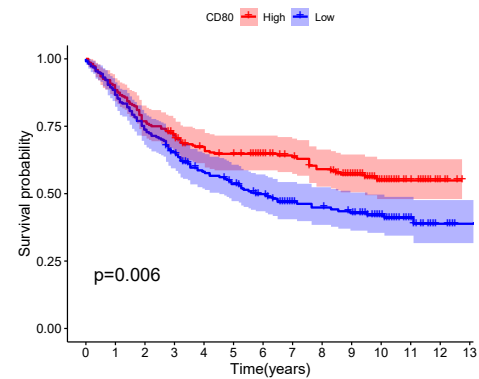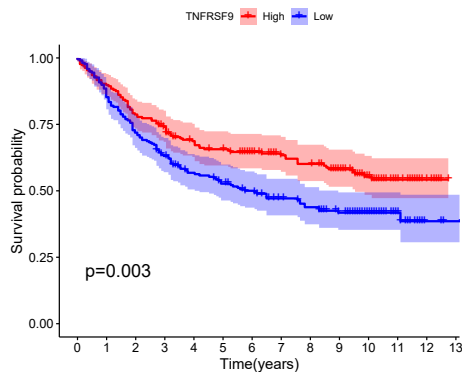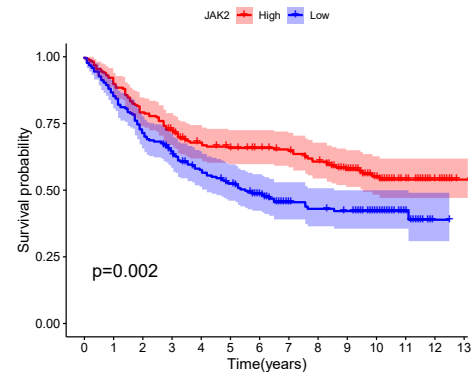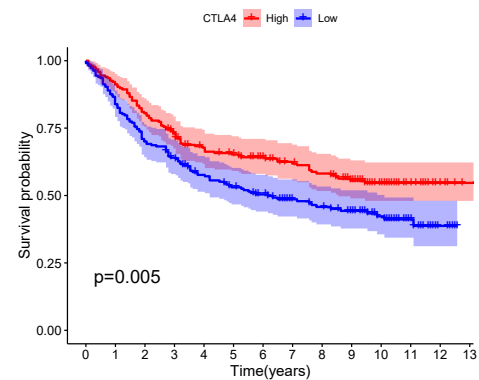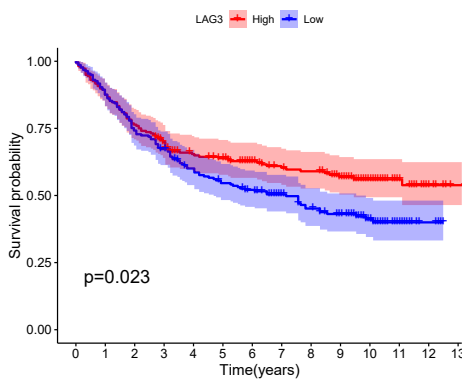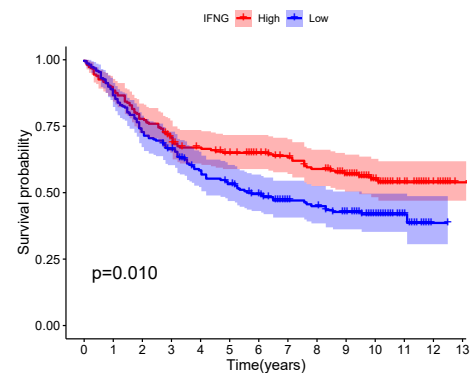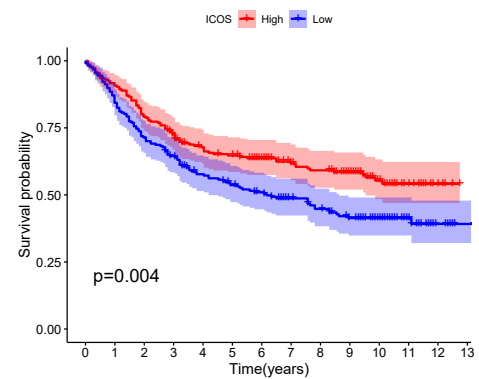

Supplement: Supplementary file 5 — Supplementary Figure 5. [file 41598_2023_38822_MOESM5_ESM.pdf]

A

WGCNA

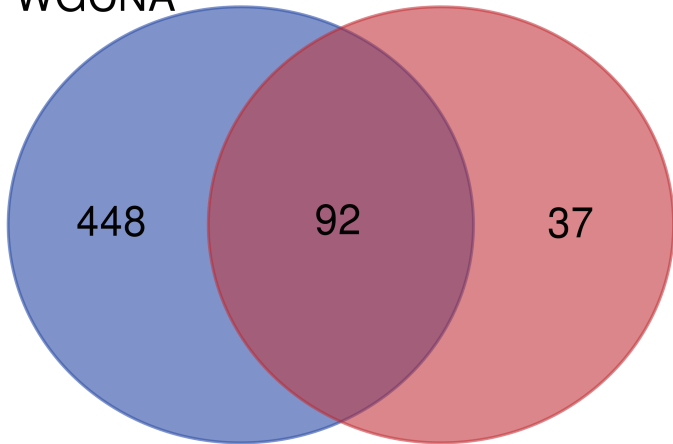

uniCOX

Supplement: Supplementary file 6 — Supplementary Figure 6. [file 41598_2023_38822_MOESM6_ESM.pdf]

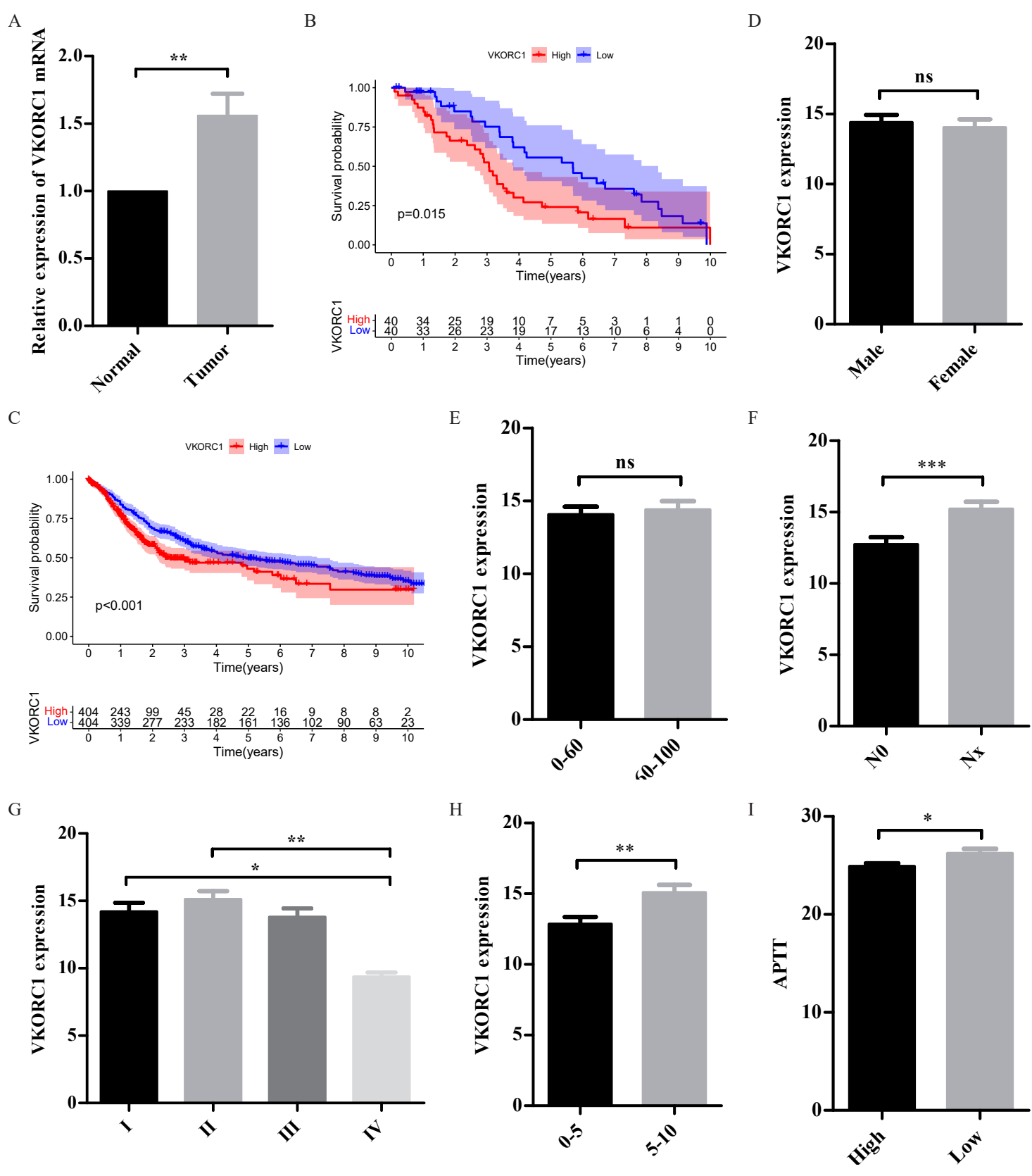

Supplement: Supplementary file 7 — Supplementary Figure 7. [file 41598_2023_38822_MOESM7_ESM.pdf]

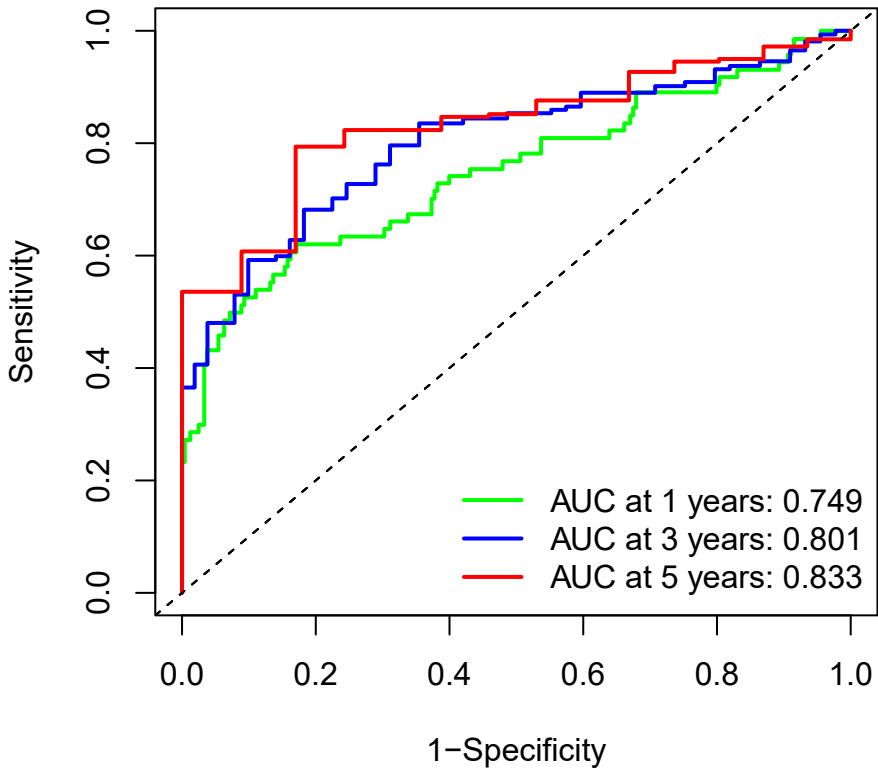

Supplement: Supplementary file 8 — Supplementary Figure 8. [file 41598_2023_38822_MOESM8_ESM.pdf]
