# Supplementary material for: Multi-omics characteristics of tumor-associated macrophages in the tumor microenvironment of gastric cancer and their exploration of immunotherapy potential
Source: Sci Rep. 2023 Oct 25;13:18265. doi: 10.1038/s41598-023-38822-2 (PMC10600170; doi:10.1038/s41598-023-38822-2)
Supplement: Supplementary file 9 — Supplementary Legends. [file 41598_2023_38822_MOESM9_ESM.docx]

**Multi-omics characteristics of tumor-associated macrophages in the tumor microenvironment of gastric cancer and their exploration of immunotherapy potential**

**Feng Cao^1,2†^, Yanwei Liu^2†^, Yunsheng Cheng^2^, Yong Wang^2*^, Yan He****^2*^, Yanyan Xu^2*^**

^1^Department of Surgery, University Hospital RWTH Aachen, Aachen, 52074, Germany.

^2^Department of General Surgery, The Second Hospital of Anhui Medical University, Hefei, 230022, China.

**Supplementary Figures Legends**

**FigureS1:** The entire analytical process of the study.

**FigureS2:** Preprocessing of the single-cell RNA sequencing data. (A-B) Gene filtering and the correlation between UMIs and mitochondrial ratio and the total number of genes.

**FigureS3:** Enrichment and unsupervised clustering analysis in GEO datasets. (A-B) GO and KEGG enrichment analyses of MDMs. (C) Unsupervised clustering of MDMs and consensus matrix heatmaps for k = 2 base on GEO datasets.

**FigureS4:** Immune cell analysis associated with prognosis.

**FigureS5:** Immune checkpoint analysis associated with prognosis.

**FigureS6:** Venn diagram of prognostic associated genes by WGCNA and univariate COX regression analysis.

**FigureS7:** VKORC1 expression and correlation with clinical parameters. (A) RT-qPCR detection of VKORC1 mRNA expression. (B) Overall survival (OS) of GC patients in the VKORC1-high and low groups based on TCGA and GEO database. (C) OS of GC patients in the VKORC1-high and low groups based on own database. (D-H) VKORC1 expression difference in distinct clinical group. (I) APTT difference in VKORC1-high and low groups.

**FigureS8:** A ROC curve of the nomogram graph.
